# The CareFirst Patient-Centered Medical Home Program: Cost and Utilization Effects in Its First Three Years

**DOI:** 10.1007/s11606-016-3814-z

**Published:** 2016-07-29

**Authors:** Alison Cuellar, Lorens A. Helmchen, Gilbert Gimm, Jay Want, Sriteja Burla, Bradley J. Kells, Iwona Kicinger, Len M. Nichols

**Affiliations:** 1 Department of Health Administration and Policy, George Mason University, 4400 University Drive, Fairfax, VA 22030 USA; 2 Department of Health Policy and Management, George Washington University, Washington, DC USA; 3 Center for Improving Value in Health Care, Denver, CO USA; 4 Center for Health Policy Research and Ethics, George Mason University, Fairfax, VA USA

**Keywords:** patient-centered care, primary care redesign, program evaluation

## Abstract

**Background:**

Enhanced primary care models have diffused slowly and shown uneven results. Because their structural features are costly and challenging for small practices to implement, they offer modest rewards for improved performance, and improvement takes time.

**Objective:**

To test whether a patient-centered medical home (PCMH) model that significantly rewarded cost savings and accommodated small primary care practices was associated with lower spending, fewer hospital admissions, and fewer emergency room visits.

**Design:**

We compared medical care expenditures and utilization among adults who participated in the PCMH program to adults who did not participate. We computed difference-in-difference estimates using two-part multivariate generalized linear models for expenditures and negative binomial models for utilization. Control variables included patient demographics, county, chronic condition indicators, and illness severity.

**Participants:**

A total of 1,433,297 adults aged 18–64 years, residing in Maryland, Virginia, and the District of Columbia, and insured by CareFirst for at least 3 consecutive months between 2010 and 2013.

**Intervention:**

CareFirst implemented enhanced fee-for-service payments to the practices, offered a large retrospective bonus if annual cost and quality targets were exceeded, and provided information and care coordination support.

**Measures:**

Outcomes were quarterly claims expenditures per member for all covered services, inpatient care, emergency care, and prescription drugs, and quarterly inpatient admissions and emergency room visits.

**Results:**

By the third intervention year, annual adjusted total claims payments were $109 per participating member (95 % CI: −$192, −$27), or 2.8 % lower than before the program and compared to those who did not participate. Forty-two percent of the overall decline in spending was explained by lower inpatient care, emergency care, and prescription drug spending. Much of the reduction in inpatient and emergency spending was explained by lower utilization of services.

**Conclusions:**

A PCMH model that does not require practices to make infrastructure investments and that rewards cost savings can reduce spending and utilization.

**Electronic supplementary material:**

The online version of this article (doi:10.1007/s11606-016-3814-z) contains supplementary material, which is available to authorized users.

## INTRODUCTION

Numerous models have been proposed for enhancing primary care and improving care coordination, while pursuing the triple aim of greater access, lower costs, and improved quality. These models range from patient-centered medical homes (PCMH) to accountable care organizations (ACOs).^1^ Many small physician practices, which provide most of the primary care services delivered in the United States, struggle to meet the requirements of even a standard PCMH model, citing large investments in infrastructure such as electronic medical records, retraining, workflow redesign, ongoing certification, and additional care coordination personnel, which can cost up to $100,000 per physician by some estimates.^2^^–^ 4 Some observers have argued that policy initiatives aimed at promoting these models could unintentionally lead to greater consolidation of physician practices and spell the end of small-scale practices.^5^

Most PCMH programs to date have relied on per-member per-month (PMPM) case management fees to finance the additional resources needed.^6^^–^ 9 While such models are suited to both large and small practices, they may not be sufficient to cover the increased practice costs necessary to perform PCMH functions or explicitly reward performance. In at least one PCMH program, practices were given access to additional staffing from a community health team, potentially benefitting smaller practices.^10^ Other PCMH programs have required third-party PCMH accreditation or have paid practices up front to meet certification criteria as a PCMH.^11^^–^ 13 Practices that do not receive financial support to become PMCH-certified are otherwise disadvantaged.

The Comprehensive Primary Care initiative (CPCI) required substantial PMPM payments from multiple payers, and offered shared savings based on quality and cost performance, but was not limited to practices with PCMH recognition.^14^ The initiative required changes in care delivery to enhance access, care planning, chronic care management, care coordination, and patient engagement. Despite some initial promising results, in the second year practices on average showed no savings in Medicare spending after accounting for the PMPM.^15^

Other PCMH initiatives have relied on modified fee-for-service payments that embed quality and spending incentives.^16^^–^ 21 Still other initiatives have relied on global budgets and “two-sided” financial risk, meaning that practices face the prospect of financial reward or penalty, depending on whether spending targets—and potentially quality targets—are or are not met.^22^ One recent example is the Massachusetts Alternative Quality Contract (AQC) program, which tied rewards to both quality metrics and spending targets, and which was directed at larger multispecialty groups or integrated systems that were in a strong position to bear financial risk.^23^ Small primary care practices, however, are not able to take on the same financial risk as large practices.

We studied the CareFirst BlueCross BlueShield’s PCMH program, which began in 2011, and has over 1 million enrollees in Maryland, the District of Columbia, and northern Virginia as of 2016. In contrast to other PCMH programs nationwide, the CareFirst program did not require large up-front investments by participating practices, a feature that made the program particularly well suited for adoption by small, independent practices. By 2013, just over 4000 primary care physicians and nurse practitioners had joined the program, representing more than 81 % of all primary care providers in the plan’s networks in Maryland, northern Virginia, and DC.^24^ This number of participating primary care physicians compares favorably with the 2222 providers nationwide who have participated in the CPCI program.

### Study Setting

For statistical validity, CareFirst grouped smaller practices together to create “panels” or clusters of approximately 10 physicians. Because performance was measured and rewarded at the panel level, each practice had an incentive to communicate with other practices in the same panel. Primary care practices with more than 20 physicians were subdivided into panels of 10. From 2011 to 2013, the number of participating physicians grew steadily, from 3476 to 4037, while the number of attributed CareFirst members increased from 987,000 to 1,169,000.

Importantly, the program did not require external certification by a PCMH-accrediting organization, although it did contain the core PCMH attributes defined by AHRQ: comprehensive and coordinated care through an array of nurse coordinators, along with hospital transition, chronic, complex, behavioral, and substance abuse care managers; accessible services through same-day appointments and 24/7 phone triage; patient-centeredness through care plans developed by nurses, clinicians, and patients together; and quality through objective performance metrics required for earning shared savings.^25^

CareFirst offered participating practices a one-time 12 % increase in fee-for-service payments for services provided by the practices immediately upon joining the program, which averaged to approximately $10.34 per member per month. Panels were not put at financial risk, but were offered additional financial rewards—up to 80 % of annual fee-for-service billings—depending on their joint quality of care and spending growth performance each year. In addition, providers could receive separate payments for developing and maintaining care plans for selected patients. The insurer provided nurse coordinators and lists of members likely to benefit from care coordination. Nurse coordinators and physicians identified and focused on subsets of each panel’s 50 most severely ill patients. The nurse coordinators developed care plans, coordinated with families, and provided follow-up support.

CareFirst also provided an electronic portal through which panels could monitor their financial and claims-based quality performance and compare the efficiency of referrals across specialists and hospitals. The detailed information captured in the portal was based on members’ medical claims with a 1-month lag so that providers could track their cost performance continuously throughout the year. Using this portal, physicians were able not only to view information on specialist costs to inform referrals, but also to obtain patient-level reports to identify gaps in care and review care plans, with notes written by care coordinators with input from providers engaged in a patient’s care.

About 70 % of eventually participating members were attributed to participating panels in 2011, the first year of operation. Thus, the program was not fully implemented at a single starting point. In addition to delays in physician participation, program features were rolled out over the first 2 years. First, nurse care coordinators had to contact roughly 400 participating panels, an effort that was hampered by high initial rates of turnover in care coordinator staff. Second, the electronic physician portal was introduced in 2012, and was underutilized until program consultants were hired to assist practices in the use of the performance data through the portal. For these reasons, our evaluation of the CareFirst PCMH program is best understood as an effectiveness study of a large-scale program that was faced with the usual challenges of real-world implementation.

We examined whether a member’s attribution to a participating PCMH panel was associated with lower total payments and lower payments for inpatient care, emergency department visits, and prescription drugs. Given the program’s focus, we hypothesized that its impact on payments would be larger for patients with chronic conditions. We also tested whether the program was associated with reductions in inpatient admissions and emergency room visits. We evaluated results in all 3 years of the program’s operation. We focus our discussion on outcomes in the third year, since the literature has shown that PCMH programs typically take a few years to reach maturity and produce measurable effects.

## METHODS

### Study Population

The study population included all adults aged 18 to 64 years who were covered by CareFirst for at least 3 consecutive months between 2010 and 2013. Individuals were included in the analysis if CareFirst held their medical and prescription drug claims. Individuals who had prescription drug coverage outside CareFirst were excluded. Monthly claims data were collapsed to quarterly observations to smooth monthly fluctuations but still capture seasonal trends. Online Appendix 1 illustrates our sample construction. The study was approved by the George Mason University institutional review board.

### Definition of Intervention and Comparison Groups

Practices were able to join the program beginning January 1, 2011. Insured members were attributed to the participating primary care panel considered most responsible for that member’s primary care, based on the previous 12 months of evaluation and management claims office and preventive care visits in an outpatient setting.

### Data

Medical and prescription drug claims data were provided by CareFirst. For each member and quarter, we summed the allowed amounts for medical and prescription drug claims. We also included members’ out-of-pocket payments. We calculated quarterly allowed amounts separately for inpatient care, emergency department visits, and prescription drug claims. In addition, we calculated the number of emergency department visits and inpatient admissions per member-quarter. Chronic conditions were measured using diagnoses in the claims data. The illness burden was measured as a prospective risk score using DxCG Intelligence software (Verisk Health, Waltham, MA) based on the previous 12 months of claims, and was provided for each member-month by the insurer.^26^

### Statistical Approach

The member-quarter was our unit of analysis. The primary dependent variable was the total claims allowed amount. We used a difference-in-differences estimator to capture changes in participant spending relative to changes in non-participant spending. We addressed observed differences between treatment and comparison members with treatment-on-the-treated propensity score weighting. The weighting models predicted the probability of being in the treatment group in the base year as a function of demographic characteristics, whether the covered individual was an employee or dependent, group size, whether the individual had a chronic condition, and illness burden. In addition to these covariates, all models included quarter and county fixed effects.

We also weighted each year of treatment and control observations to the baseline year for the treatment group in order to control for any compositional changes over time.^27^ The weighting models were estimated using boosted regression, as implemented in the "twang" package in R.^28^ We estimated two-part multivariate generalized linear models with a log link and gamma distribution to isolate the association between a member’s attribution to a participating primary care practice and quarterly spending.^29^ For inpatient admissions and emergency room visits, we estimated zero-inflated negative binomial or hurdle models with the same set of control variables. We clustered standard errors at the panel level.

Members who were continuously attributed to a participating panel were defined as the intervention group. We refer to this group as “always PCMH” (*N* = 592,886 individuals). Because some physician panels joined the program as early as January 2011, a member could be attributed to participating practices for as many as 3 years during our study period. Thus, we measured the association of spending with program participation in the first, second, and third years. Some members were ineligible for attribution, either because their primary care provider was in a non-participating practice or because their employer declined to have its employee members participate in the program. The members who were never attributed to the PCMH model during our study period constituted the comparison group.

### Robustness Checks

As a robustness check, we defined a second, more expansive intervention group of members who were attributed for at least one quarter, but may not have been continuously attributed to a participating panel thereafter. This second group, referred to as “ever PCMH” (*N* = 804,758 individuals), included individuals with less exposure to the PCMH program than the “always PCMH” group.

## RESULTS

The characteristics of members in the “always PCMH” intervention group were similar to those in the treatment group in the baseline year after weighting (Table 1). The covariate balance from propensity score weighting across all years, as measured by the standardized mean differences for each pair of covariates, is shown in Online Appendix 2. Balance was achieved with all 100 covariate pairs having a standardized difference of less than 0.10. Continuously attributed members, the “always PCMH” intervention group, had lower spending in the baseline quarter than the comparison group of members who were never attributed (Table 1; $966 vs. $1107, *p* < 0.001). Expenditures for prescription drugs ($131 vs. $108, *p* < 0.001) were higher, but emergency room ($44 vs. $46, *p* = 0.039) and inpatient care ($114 vs. $135, *p* < 0.001) were lower.

**Table 1:** CareFirst Enrollee Descriptive Characteristics – 2010 Quarter 1, Propensity Score-Weighted

	Comparison mean (95 % CI)	Ever PCMH mean (95 % CI)	*p* value	Always PCMH mean (95 % CI)	*p* value
Total allowed amount ($)	1107 (1082–1131)	1000 (980–1021)	<0.001	966 (946–987)	<0.001
Inpatient allowed amount ($)	135 (127–142)	120 (112–128)	0.007	114 (106–121)	<0.001
ED allowed amount ($)	46 (44–48)	46 (44–47)	0.672	44 (42–45)	0.039
Rx allowed amount ($)	108 (106–109)	130 (129–132)	<0.001	131 (129–133)	<0.001
Inpatient admissions per 1000	146 (143–149)	135 (132–139)	<0.001	129 (126–133)	<0.001
ER visits per 1000	21 (20–21)	20 (19–21)	0.154	19 (18–20)	0.007
Age (years)	44 (44–44)	43 (43–43)	<0.001	43 (43–43)	<0.001
Female (%)	53 (52–-53)	58 (57–58)	0.545	56 (56–56)	0.519
Illness burden score	88 (88–89)	92 (91–93)	<0.001	89 (88–90)	0.126
Fully insured group (%)	45 (45–45)	44 (44–45)	0.034	44 (44–45)	0.017
Employee (%)	67 (67–67)	66 (66–66)	<0.001	67 (67–-67)	0.121
Small employer (%)	16 (16–16)	17 (17–17)	<0.001	17 (16–17)	0.014
No chronic conditions (%)	39 (39–40)	39 (39–39)	0.029	39 (39–39)	0.034
One chronic condition (%)	25 (25–26)	25 (25–25)	<0.001	25 (25–25)	0.116
Two or more chronic conditions (%)	35 (35–35)	36 (36–37)	<0.001	36 (36–36)	<0.001
Maryland resident (%)	44 (44-44)	79 (79–79)	<0.001	79 (79–79)	<0.001
Member count (unweighted)	628,539	804,758		592,886	

*ED* emergency department, *Rx* prescription drug

**Figure 1:**
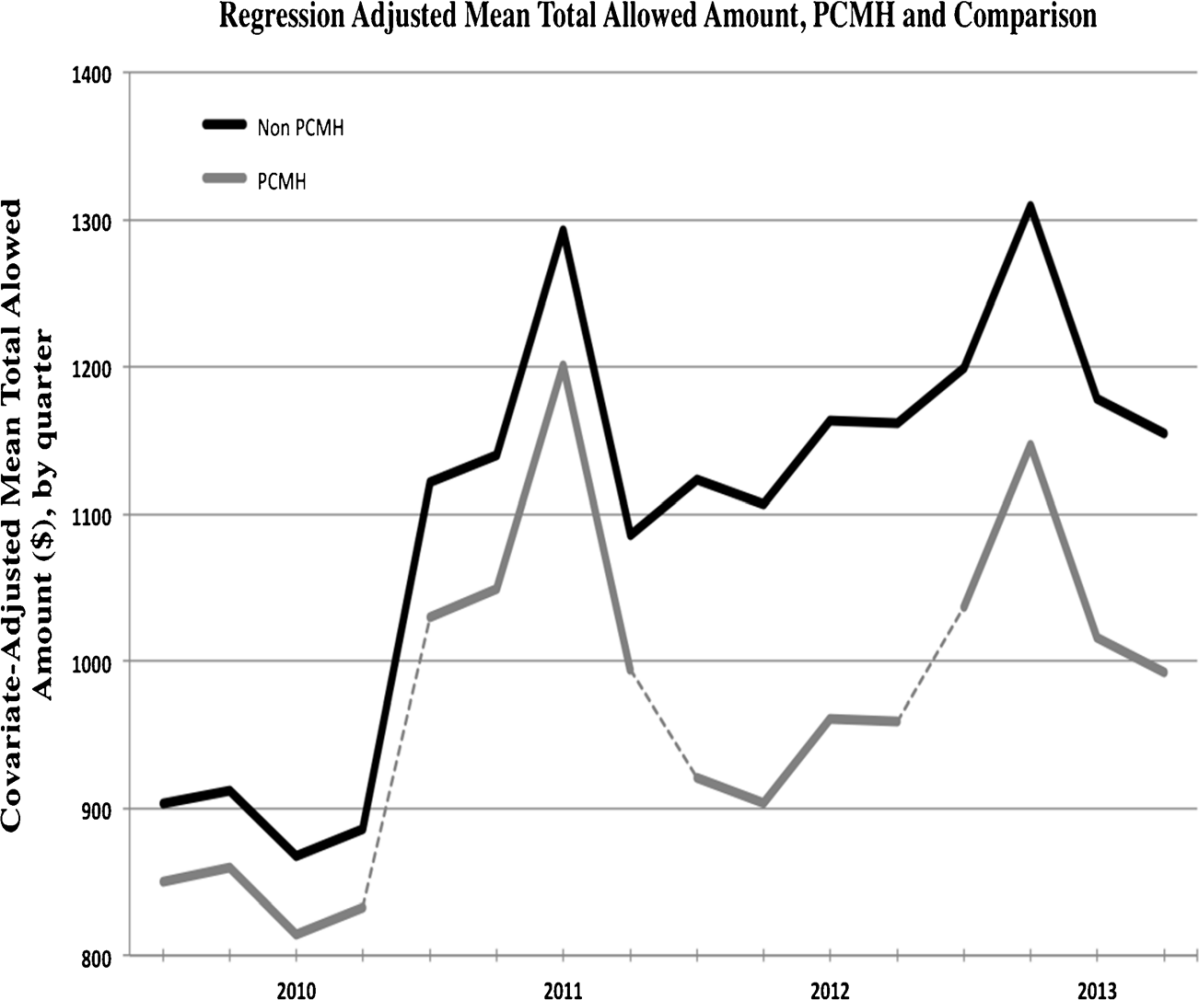
Regression-adjusted mean total allowed amount, PCMH, and comparison

Continuously attributed members recorded lower expenditures by the second and third years relative to the comparison group (Table 2). There were no statistically significant differences in total expenditures between the intervention and comparison groups in the first program year. For the third year, we estimated a reduction in total spending per member of $109 (95 % CI: −$191.82, −$26.96), equivalent to a decline of 2.8 % relative to base year. The total 3-year savings was $297 (95 % CI: −471.41, −123.69) per PCMH participant relative to comparisons. Figure 1 illustrates the regression-adjusted means for both the treatment and comparison groups for all 4 years (baseline and the 3 intervention years). Full regression results for the expenditure models are provided in Online Appendix 3.

**Table 2:** Total Expenditures – Annual Marginal Effects

	All members		Members with chronic conditions
Year-on-year change in total allowed amount ($)	Always PCMH	Ever PCMH	Always PCMH
Intervention year 1	−38.4	−16.3	−58.3
	[−105.83, 29.09]	[−69.13, 36.44]	[−183.06, 66.41]
Intervention year 2	−149.8^†^	−126.57^‡^	−235.9^‡^
	[−201.32, −98.26]	[−144.95, −108.20]	[−344.22, −127.53]
Intervention year 3	−109.4^‡^	−159.9^‡^	−143.7^†^
	[−191.82, −26.96]	[−182.18, −137.72]	[−250.04, −37.42]
Member-quarters	21,008,072	24,641,055	10,459,946
2010 Annualized spending per member ($)*	3864	4000	5080

Two-part multivariate models controlling for: quarter and county fixed effects, age, gender, number of chronic conditions, illness burden, fully insured group, dependent status, employer size, and county

* Calculated as 4 times the intervention group’s first quarter spending in 2010

95 % confidence intervals in brackets

^†^ *p* <0.01, ^‡^ *p* <0.001

We estimated analogous average reductions for year 3 of $23 in inpatient spending (95 % CI: −$35, −$11), $8 in emergency department spending (95 % CI: −$11, −$5), and $14 in prescription drug spending (95 % CI: −$20, −$9) (Table 3). The percentage reductions relative to 2010 were 5.0 % for inpatient care, 4.5 % for emergency care, and 2.7 % for prescription drugs.

**Table 3:** Inpatient, Emergency Room, and Drug Expenditures – Annual Marginal Effects

	IP allowed amount		ER allowed amount		Rx allowed amount	
	Always PCMH	Ever PCMH	Always PCMH	Ever PCMH	Always PCMH	Ever PCMH
Intervention year1	−9.6^†^	−10.6^†^	−1.3	−0.7	2.0	−0.3
	[−16.83, −2.39]	[−17.35, −3.94]	[−3.49, 0.81]	[−2.72, −1.40]	[−3.47, 7.45]	[−6.81, 6.13]
Intervention year2	−25.8^‡^	−23.3^‡^	−6.1^‡^	−5.7^‡^	−10.5^‡^	−10.2^‡^
	[−34.10, −17.41]	[−29.87, −16.82]	[−8.48, −3.69]	[−8.09, −3.28]	[−15.20, −5.83]	[−15.19, −5.17]
Intervention year3	−23.0^‡^	−26.4^‡^	−8.3^‡^	−8.3^‡^	−14.3^‡^	−17.4^‡^
	[−34.96, −11.08]	[−35.06, −17.87]	[−11.12, −5.41]	[−11.42, −5.19]	[−19.63, −9.00]	[−22.51, −12.38]
Observations	21,008,072	24,641,055	21,008,072	24,641,055	21,008,072	24,641,055
2010 Annualized spending per member ($)*	456	480	176	184	516	520

Two-part multivariate models controlling for: quarters by year, age, gender, no. of chronic conditions, illness burden, fully insured group, dependent status, employer size, and county

* Calculated as 4 times the intervention group’s first quarter spending in 2010

95 % confidence intervals in brackets

^†^ *p* <0.01, ^‡^ *p* <0.001

ED emergency department, Rx prescription drug

Among individuals with chronic conditions (Table 2), the absolute reduction in total spending in year 3 was greater than that for all members ($144 vs. $109), but as a percentage of annualized spending it was equivalent (2.8 %). Twenty percent of the total reduction was due to inpatient spending, which declined by $32 (CI: −$56, −$9) by year 3 (Table 4). Also, by year 3, reductions in emergency room spending were larger for individuals with chronic conditions than for all individuals ($10 vs. $8), as was prescription drug spending ($18 vs. $14); neither difference was statistically significant between PCMH participants with chronic conditions and all participants.

**Table 4:** Inpatient, Emergency Room, and Drug Expenditures, Annual Marginal Effects – Chronic Group Only

	IP allowed amount	ER allowed amount	Rx allowed amount
	Always PCMH	Always PCMH	Always PCMH
Intervention year1	−7.9	−1.8	4.7
	[−21.63,5.80]	[−4.89, 1.30]	[−3.47, 7.45]
Intervention year2	−35.8^‡^	−7.3^‡^	−12.2^†^
	[−52.50, −19.01]	[−10.9, -3.81]	[−20.11, −4.22]
Intervention year3	−32.0^†^	−9.9^‡^	−17.8^‡^
	[−55.59, −8.50]	[−14.12, −5.71]	[−26.79, −8.75]
Observations	10,459,946	10,459,946	10,459,946
2010 Annualized spending per member ($)*	704	208	679

Two-part multivariate models controlling for: quarters by year, age, gender, no. of chronic conditions, illness burden, fully insured group, dependent status, employer size, and county

* Calculated as 4 times the intervention group’s first quarter spending in 2010

95 % confidence intervals in brackets

^†^ *p* <0.01, ^‡^ *p* <0.001

ED emergency department, Rx prescription drug

The program was associated with reductions in inpatient admissions by the third year (Table 5). In year 3, members experienced 2.4 (95 % CI: −2.8, −2.2) fewer inpatient admissions per 1000 on average, representing a 2.4 % reduction. They also had 9.9 (95 % CI: −9.0, −7.7) fewer emergency room visits per 1000 in year 3, a decline of 3.2 %. Full regression results for the utilization models are provided in Online Appendix 4.

**Table 5:** Inpatient Admissions and Emergency Room Visits – Annual Marginal Effects

	IP admissions (per 1000)	ER visits (per 1000)
	Always PCMH	Always PCMH
Intervention year1	−0.55	−2.27
	[−1.37, −0.28]	[−5.59, 1.05]
Intervention year2	−2.43^‡^	−8.36^‡^
	[−3.28, −1.56]	[−11.62, −5.11]
Intervention year3	−2.35^‡^	−9.94^‡^
	[−3.38, −1.31]	[−14.33, −5.54]
Observations	21,008,072	21,008,072
2010 Annualized IP admissions/ER visits per 1000*	96	312

Zero-inflated negative binomial models controlling for: quarters by year, age, gender, no. of chronic conditions, illness burden, fully insured group, dependent status, employer size, and county

*Calculated as 4 times the intervention group’s first quarter amounts in 2010

95 % confidence intervals in brackets

^†^ *p* <0.01, ^‡^ *p* <0.001

ED emergency department, Rx prescription drug

### Robustness Results

When we expanded the intervention group to also include “ever” members who were attributed to participating practices only intermittently, the estimated impact of the program was in the same direction but larger in magnitude than in the main models using “always” participants. “Ever” robustness results are shown in column 2 of Table 2 for total allowed amounts, with full results in Online Appendix 5.

## DISCUSSION

Implementation of the CareFirst PCMH program was associated with lowering of costs by year 2, and 2.8 % lower total payments by year 3. This compares favorably to most early PCMH programs with quality and spending incentives, which observed small or no effects on spending.^30^ Other PCMH programs have also been shown to reduce overall expenditures, inpatient care, or emergency room care, but they required meeting the full catalogue of PCMH accreditation criteria or substantial up-front investments, which are particularly onerous for small physician practices.^31^^–^ 33

The magnitude of the reduction was greatest for members with chronic conditions, consistent with other studies of coordinated care interventions.^34^ The gross decline in spending is comparable to that of the AQC program.^35^ By comparison, CMMI’s combined CPCI demonstrations lowered payments for medical services and/or utilization in some regions in year 1, but had no statistically measurable effects on cost or use on average in year 2. Since CareFirst’s incentive payments were offered as fee-for-service enhancements, they were captured by the claims data and spending calculations used in our analysis. Therefore, the results we report should be construed as net of participation fees. However, we do not have data on the amount spent by CareFirst on the information and care coordination infrastructure to implement the program. Our estimates suggest that it did reduce medical spending compared to a control group by year 2 of implementation.

The one region in the CPCI demonstration that experienced reductions in net spending in year 1 also experienced reductions in quality. Our study has not yet examined changes in quality, but minimum thresholds of quality performance—as measured mostly by claims data—were required for shared savings bonuses to be awarded by CareFirst.

In contrast to the Massachusetts AQC intervention, which was also associated with reductions in spending, 40 % of the overall decline in spending in the CareFirst program is explained by reductions in inpatient care, emergency care, and prescription drugs.

In our study, much of the reduction in inpatient and emergency care was explained by lower utilization of these services, indicating that the program may have succeeded in encouraging primary care physicians to manage both admissions and emergency visits. This could be due to lower volume of service, shifts to lower-priced settings, lower prices from acute care providers worried about volume, or lower intensity of services conditional on an admission or visit as a result of more conservative practice styles of referred specialists.

## CONCLUSION

Early experience shows that an intervention aimed at realigning primary care practice incentives could be effective in curbing spending growth and utilization. The intervention studied here is noteworthy in that it avoided burdening participating practices with the costly infrastructure investments and short-term downside risk that many other PCMH interventions have. As such, the type of intervention studied here should appeal to small practices in particular. Moreover, these results suggest that some particular structural PCMH elements may not be required for good results, which is a lesson that could inform alternative payment models by other payers, such as Medicare.

Total spending declined more than the sum of reductions in inpatient care, emergency room care, and prescription drugs. It is possible that these extra reductions could be explained by other covered services, including outpatient specialty care, laboratory tests, imaging, and home care, or by lower prices. Lower spending on outpatient specialty care would point to the possibility that referral management was an important contributor to the results reported here. The physician portal offered by this program allowed primary care physicians to identify less expensive specialists more easily. Future work should address specialty care referral outcomes and quality outcomes.

## Electronic supplementary material

Below is the link to the electronic supplementary material.

Online Appendix 1 (DOCX 51 kb)

Online Appendix 2 (DOCX 32 kb)

Online Appendix 3 (DOCX 34 kb)

Online Appendix 4 (DOCX 28 kb)

Online Appendix 5 (DOCX 38 kb)
