# Supplementary material for: The CareFirst Patient-Centered Medical Home Program: Cost and Utilization Effects in Its First Three Years
Source: J Gen Intern Med. 2016 Jul 29;31(11):1382–8. doi: 10.1007/s11606-016-3814-z (PMC5071295; doi:10.1007/s11606-016-3814-z)
Supplement: Supplementary file 1 — (DOCX 51 kb) [file 11606_2016_3814_MOESM1_ESM.docx]

**Appendix 1: Sample Construction**

Exclude:
All member-quarters with more than $2 million in claims in a quarter
(2 member-quarters)

24,631,055 member-quarters

Exclude:
All member-quarters in groups without Rx claims in that quarter
(1,072,709 member-quarters)

Exclude:
Members with 2010 observations only
(680,559 member-quarters)

24,631,057 member-quarters

Exclude:
Member-quarters without enrollment in all three months
(1,512,985 member-quarters)

Exclude:
Members younger than 19
Members older than 64
(14,141,217 member-quarters)

25,713,766 member-quarters

123,020,681 member-months

26,394,325 member-quarters

42,048,527 member-quarters

27,907,310 member-quarters

Collapse to quarter
