# Supplementary material for: The CareFirst Patient-Centered Medical Home Program: Cost and Utilization Effects in Its First Three Years
Source: J Gen Intern Med. 2016 Jul 29;31(11):1382–8. doi: 10.1007/s11606-016-3814-z (PMC5071295; doi:10.1007/s11606-016-3814-z)
Supplement: Supplementary file 2 — (DOCX 32 kb) [file 11606_2016_3814_MOESM2_ESM.docx]

**Appendix 2:**

**A) Always PCMH members: Distribution of standardized differences in means for all variables comparing non-baseline years to the baseline year, unweighted and weighted**

**B) Comparison Members: Distribution of standardized differences in means for all variables comparing each year to the baseline year for the “always PCMH” treated members, unweighted and weighted**
