# Supplementary material for: The CareFirst Patient-Centered Medical Home Program: Cost and Utilization Effects in Its First Three Years
Source: J Gen Intern Med. 2016 Jul 29;31(11):1382–8. doi: 10.1007/s11606-016-3814-z (PMC5071295; doi:10.1007/s11606-016-3814-z)
Supplement: Supplementary file 3 — (DOCX 34 kb) [file 11606_2016_3814_MOESM3_ESM.docx]

**Appendix 3: Total, Inpatient, Emergency Department, & Prescription Drug Allowed Amount –Two Part Models**

|  | **I. Total Allowed Amount** | | **II. Inpatient Allowed Amount** | | **III. Emergency Department Allowed Amount** | | **IV. Prescription Drug Allowed Amount** | |
| --- | --- | --- | --- | --- | --- | --- | --- | --- |
|  | **Logit Regression**  **(Part One)** | **Generalized Linear Model**  **(Part Two)** | **Logit Regression**  **(Part One)** | **Generalized Linear Model**  **(Part Two)** | **Logit Regression**  **(Part One)** | **Generalized Linear Model**  **(Part Two)** | **Logit Regression**  **(Part One)** | **Generalized Linear Model**  **(Part Two)** |
| PCMH | -0.004 | -0.157** | -0.023*** | -0.031* | 0.028 | -0.023** | 0.019 | -0.069*** |
|  | [-0.024, 0.015] | [-0.096, -0.017] | [-0.054, -0.036] | [-0.066, 0.004] | [-0.22, 0.78] | [-0.043, -0.004] | [-0.017, 0.056] | [-0.098, -0.040] |
| Post_Y1 | 0.146*** | -0.070* | -0.009** | -0.065** | -0.020 | -0.011 | 0.178*** | -0.070*** |
|  | [0.110, 0.182] | [-0.144, 0.044] | [-0.021, 0.002] | [-0.106, -0.024] | [-0.066, -0.027] | [-0.028, 0.007] | [0.128, 0.228] | [-0.109, -0.030] |
| Post_Y2 | 0.008 | -0.176*** | -0.08** | -0.120*** | -0.119*** | -0.026** | 0.055** | -0.120*** |
|  | [-0.025, 0.041] | [-0.239, -0.113] | [-0.094, -0.067] | [-0.178, -0.061] | [-0.175, -0.063] | [-0.046, -0.007] | [0.003, 0.106] | [0.155, 0.044] |
| Post_Y3 | 0.027 | -0.131** | -0.09** | -0.100** | -0.149*** | -0.053*** | -0.001 | -0.129*** |
|  | [-0.011, 0.066] | [-0.231, -0.031] | [-0.107, -0.074] | [-0.193, -0.009] | [-0.220, -0.078] | [-0.078, -0.028] | [-0.065, 0.063] | [-0.167, -0.090] |
| Y2010Q2 | -0.175*** | 0.046*** | -0.006** | 0.054* | 0.035** | -0.028** | 0.017** | 0.026** |
|  | [-0.223, -0.127] | [0.029, 0.062] | [-0.024, 0.012] | [-0.008, 0.117] | [0.004, 0.066] | [-0.048, -0.008] | [0.006, 0.028] | [-0.008, 0.044] |
| Y2010Q3 | -0.450*** | 0.059*** | -0.046** | 0.119*** | 0.007 | -0.012 | -0.073*** | 0.020 |
|  | [-0.496, -0.404] | [0.033, 0.085] | [-0.064, -0.028] | [0.050, 0.187] | [-0.024, 0.038] | [-0.040, 0.015] | [-0.095, -0.050] | [-0.006, 0.046] |
| Y2010Q4 | -0.534*** | 0.100*** | -0.045** | 0.150*** | -0.046** | 0.018* | -0.078*** | 0.027** |
|  | [-0.588, -0.480] | [0.081, 0.120] | [-0.063, -0.028] | [0.099, 0.201] | [-0.078, -0.015] | [-0.003, 0.039] | [-0.121, -0.035] | [0.009, 0.045] |
| Y2011Q1 | -0.370*** | 0.295*** | 0.052** | 0.272*** | 0.083*** | 0.062*** | 0.015 | 0.071** |
|  | [-0.418, -0.322] | [0.169, 0.421] | [0.033, 0.07] | [0.159, 0.385] | [0.042, 0.124] | [0.037, 0.087] | [-0.010, 0.039] | [0.017, 0.124] |
| Y2011Q2 | -0.136*** | 0.260*** | 0.049** | 0.234*** | 0.086*** | 0.044** | -0.010 | 0.105*** |
|  | [-0.167, -0.105] | [0.136, 0.383] | [0.031, 0.068] | [0.165, 0.302] | [0.051, 0.122] | [0.014, 0.075] | [-0.030, 0.010] | [0.049, 0.160] |
| Y2011Q3 | 0.072* | 0.346*** | 0.059** | 0.307*** | 0.155** | 0.080*** | -0.058*** | 0.137*** |
|  | [-0.000, 0.144] | [0.160, 0.532] | [0.04, 0.077] | [0.198, 0.415] | [0.099, 0.211] | [0.052, 0.107] | [-0.082,-0.033] | [0.068, 0.205] |
| Y2011Q4 | 0.374* | 0.176** | -0.066** | 0.285*** | 0.057** | 0.065*** | -0.086*** | 0.064*** |
|  | [0.005, 0.070] | [0.062, 0.289] | [-0.085, -0.047] | [0.216, 0.355] | [0.021, 0.094] | [0.043, 0.087] | [-0.127, -0.044] | [0.027, 0.100] |
| Y2012Q1 | 0.097*** | 0.199*** | -0.039** | 0.267*** | 0.128*** | 0.114*** | -0.160*** | 0.095*** |
|  | [0.082, 0.112] | [0.130, 0.267] | [-0.057, -0.02] | [0.192, 0.342] | [0.087, 0.168] | [0.909, 0.137] | [-0.190, -0.132] | [0.070, 0.120] |
| Y2012Q2 | 0.021** | 0.198*** | -0.057** | 0.166*** | 0.126*** | 0.140*** | -0.274*** | 0.106*** |
|  | [0.006, 0.036] | [0.143, 0.253] | [-0.075, -0.038] | [0.091, 0.240] | [0. 086, 0. 166] | [0.115, 0.165] | [-0.311, -0.237] | [0.081, 0.131] |
| Y2012Q3 | 0.011 | 0.251** | -0.075** | 0.283*** | 0. 143*** | 0.167*** | -0.413*** | 0.081*** |
|  | [-0.008, 0.030] | [0.086, 0.415] | [-0.094, -0.056] | [0.149, 0.416] | [0. 091, 0. 195] | [0.143, 0.190] | [-0.459, -0.368] | [0.049, 0.112] |
| Y2012Q4 | 0.047*** | 0.242*** | -0.097** | 0.199*** | 0. 090*** | 0.166*** | -0.419*** | 0.110*** |
|  | [0.029, 0.65] | [0.195, 0.289] | [-0.116, -0.078] | [0.114, 0.284] | [0. 039, 0. 141] | [0.143, 0.190] | [-0.470, -0.370] | [0.078, 0.142] |
| Y2013Q1 | 0.050** | 0.274*** | -0.104** | 0.330*** | 0. 105*** | 0.191*** | -0.393*** | 0.138*** |
|  | [0.018, 0.081] | [0.217, 0.330] | [-0.123, -0.085] | [0.259, 0.402] | [0. 036, 0. 173] | [0.165, 0.217] | [-0.430, -0.357] | [0.116, 0.160] |
| Y2013Q2 | 0.001 | 0.373*** | -0.09** | 0.391*** | 0. 106** | 0.194*** | -0.439*** | 0. 207*** |
|  | [-0.025, 0.027] | [0.280, 0.465] | [-0.109, -0.071] | [0.295, 0.486] | [0. 045, 0. 166] | [0.174, 0.214] | [-0.484, -0.393] | [0. 1780. 235] |
| Y2013Q3 | 0.154*** | 0.236*** | -0.072** | 0.295*** | 0. 113*** | 0.254*** | -0.406*** | 0. 223*** |
|  | [0.132, 0. 176] | [0.151, 0.321] | [-0.092, -0.053] | [0.213, 0.377] | [0. 048, 0. 178] | [0.230, 0.279] | [-0.452, -0.360] | [0. 197, 0. 248] |
| Y2013Q4 | 0.244*** | 0.200*** | -0.124** | 0.272*** | 0. 038 | 0.265*** | -0.391*** | 0. 206*** |
|  | [0.213, 0.275] | [0.097, 0.303] | [-0.144, -0.105] | [0.193, 0.352] | [-0.036, 0.112] | [0.236, 0.294] | [-0.438, -0.345] | [0. 156, 0. 255] |
| Illness Burden | 0.005*** | 0.004*** | 0.01*** | 0.002*** | 0.006*** | 0.001*** | 0.003*** | 0. 003*** |
|  | [0.005, 0.005] | [0.004, 0.004] | [0.01, 0.01] | [0.001, 0.002] | [0.005, 0.006] | [0.001, 0.001] | [0.003, 0.003] | [0. 003, 0. 003] |
| Age 19-29 | 0.157*** | 0.077*** | 0.667** | 0.145*** | 0.938*** | 0.116*** | 0.224*** | -0. 133*** |
|  | [0.145, 0.169] | [0.053, 0.102] | [0.656, 0.677] | [0.098, 0.191] | [0.909, 0.967] | [0.100, 0.131] | [0.203, 0.247] | [-0. 159, -0. 107] |
| Age 30-39 | 0.124*** | 0.126*** | 0.793*** | 0.052** | 0.554*** | 0.089*** | 0.140*** | -0.146*** |
|  | [0.114, 0.134] | [0.108, 0.144] | [0.784, 0.801] | [0.007, 0.097] | [0.533, 0.575] | [0.076, 0.102] | [0.103, 0.178] | [-0.171, -0.121] |
| Age 40-49 | 0.035*** | -0.022** | 0.098*** | -0.098*** | 0.331*** | 0.075*** | 0.072*** | -0.054*** |
|  | [0.026, 0.045] | [-0.044, 0.001] | [0.089, 0.108] | [-0.142, -0.054] | [0.319, 0.343] | [0.063, 0.086] | [0.048, 0.097] | [-0.071,-0.037] |
| Male/Other | -0.549*** | -0.013 | -0.267*** | 0.160*** | 0.016** | -0.057*** | -0.371*** | 0.182*** |
|  | [-0.563, -0.534] | [-0.033, 0.066] | [-0.274, -0.261] | [0.135, 0.184] | [-0.000, 0.033] | [-0.066, -0.048] | [-0.406, -0.335] | [0.152, 0.212] |
| Risk | 0.599*** | 0.016 | -0.086*** | -0.067*** | 0.030 | 0.084*** | 2.702*** | -0.331*** |
|  | [0.541, 0.658] | [-0.005, 0.038] | [-0.094, -0.079] | [-0.105, -0.028] | [-0.006, 0.067] | [0.066, 0.101] | [2.382, 3.022] | [-0.391,-0.271] |
| 1 Condition | 0.483*** | 0.139*** | -0.146*** | 0.081*** | 0.055*** | 0.063*** | 0.542*** | 0.170*** |
|  | [0.424, 0.543] | [0.114, 0.165] | [-0.155, -0.136] | [0.045, 0.116] | [0.035, 0.074] | [0.051, 0.076] | [0.521, 0.563] | [0.128, 0.211] |
| 2+ Conditions | 0.962*** | 0.421*** | 0.047*** | 0.226*** | 0.199*** | 0.096*** | 0.866*** | 0.391*** |
|  | [0.880, 1.045] | [0.367, 0.476] | [0.038, 0.056] | [0.175, 0.277] | [0.163, 0.234] | [0.081, 0.110] | [0.842, 0.889] | [0.361, 0.421] |
| Self | 0.070*** | -0.048*** | -0.123*** | -0.026 | 0.016** | -0.035*** | 0.082*** | -0.056*** |
|  | [0.059, 0.082] | [-0.067, -0.029] | [-0.13, -0.116] | [-0.067, 0.014] | [0.003, 0.029] | [-0.048, -0.022] | [0.070, 0.094] | [-0.088,-0.024] |
| Small Employer50 | 0.167*** | 0.032*** | -0.03** | -0.033* | -0.001 | 0.013* | 0.567*** | 0.086*** |
|  | [0.105, 0.230] | [0.017, 0.046] | [-0.041, -0.019] | [-0.071, 0.005] | [-0.023, 0.022] | [-0.001, 0.029] | [0.398, 0.736] | [0.052, 0.119] |
| _cons | 0.870*** | 7.066*** | -3.872** | 8.485*** | -3.272*** | 6.537*** | -2.721*** | 6.436*** |
|  | [0.836, 0.903] | [7.035, 7.096] | [-3.891, -3.852] | [8.392, 8.577] | [-3.308, -3.235] | [6.512, 6.562] | [-2.942, -2.500] | [6.359, 6.514] |
| Observations | 21,008,072 | 21,008,072 | 21,008,072 | 21,008,072 | 21,008,072 | 21,008,072 | 21,008,072 | 21,008,072 |

Two-part models controlling for: *Quarters by year, Age, Gender, # of Chronic Conditions, Illness Burden, Fully Insured Group, Dependent Status, Employer Size, County, weighted by propensity scores*

Coefficients with 95% confidence intervals in brackets

^*^ *p* < 0.05, ^**^ *p* < 0.01, ^***^ *p* < 0.001
