# Supplementary material for: The CareFirst Patient-Centered Medical Home Program: Cost and Utilization Effects in Its First Three Years
Source: J Gen Intern Med. 2016 Jul 29;31(11):1382–8. doi: 10.1007/s11606-016-3814-z (PMC5071295; doi:10.1007/s11606-016-3814-z)
Supplement: Supplementary file 4 — (DOCX 28 kb) [file 11606_2016_3814_MOESM4_ESM.docx]

**Appendix 4: Inpatient Admissions and Emergency Room Visits –Two Part Models**

|  |  | **I. Inpatient Admissions** | | **II. Emergency Room**  **Visits** | |
| --- | --- | --- | --- | --- | --- |
|  |  | **Logit Regression**  **(Part One)** | **Negative Binomial Model**  **(Part Two)** | **Logit Regression**  **(Part One)** | **Negative Binomial Model**  **(Part Two)** |
| PCMH |  | -0.051** | -0.067 | 0.022 | -0.024 |
|  |  | [-0.084,-0.019] | [-0.135,0.001] | [-0.040,0.084] | [-0.162,0.114] |
| Post_Y1 |  | -0.067** | -0.128** | -0.029 | 0.039 |
|  |  | [-0.109,-0.026] | [-0.202,-0.053] | [-0.071,0.013] | [-0.178,0.256] |
| Post_Y2 |  | -0.136*** | 0.026 | -0.116*** | 0.116 |
|  |  | [-0.182,-0.089] | [-0.053,0.105] | [-0.173,-0.059] | [-0.060,0.293] |
| Post_Y3 |  | -0.138*** | -0.001 | -0.149*** | 0.082 |
|  |  | [-0.196,-0.080] | [-0.106,0.105] | [-0.224,-0.074] | [-0.138,0.303] |
| Y2010Q2 |  | 0.025 | 0.063 | 0.025 | -0.061 |
|  |  | [-0.036,0.086] | [-0.093,0.218] | [-0.020,0.070] | [-0.253,0.131] |
| Y2010Q3 |  | 0.020 | 0.137 | 0.044* | 0.126 |
|  |  | [-0.047,0.087] | [-0.013,0.288] | [0.000,0.088] | [-0.063,0.315] |
| Y2010Q4 |  | 0.024 | 0.134 | -0.038 | 0.006 |
|  |  | [-0.036,0.084] | [-0.041,0.308] | [-0.084,0.007] | [-0.134,0.147] |
| Y2011Q1 |  | 0.074** | -0.004 | 0.031 | -0.465 |
|  |  | [0.022,0.127] | [-0.175,0.166] | [-0.050,0.112] | [-1.271,0.341] |
| Y2011Q2 |  | 0.091** | 0.132 | 0.047 | -0.406 |
|  |  | [0.035,0.146] | [-0.041,0.304] | [-0.019,0.113] | [-1.063,0.251] |
| Y2011Q3 |  | 0.090** | 0.102 | 0.091 | -0.687 |
|  |  | [0.027,0.153] | [-0.107,0.311] | [-0.010,0.191] | [-2.022,0.648] |
| Y2011Q4 |  | 0.033 | 0.183* | 0.064*** | -0.098 |
|  |  | [-0.021,0.087] | [0.028,0.338] | [0.031,0.098] | [-0.386,0.190] |
| Y2012Q1 |  | 0.060 | 0.102 | 0.144*** | -0.043 |
|  |  | [-0.010,0.129] | [-0.046,0.251] | [0.097,0.191] | [-0.189,0.103] |
| Y2012Q2 |  | 0.069* | 0.199** | 0.137*** | -0.057 |
|  |  | [0.014,0.125] | [0.062,0.336] | [0.091,0.184] | [-0.207,0.092] |
| Y2012Q3 |  | 0.066* | 0.214** | 0.119*** | -0.275 |
|  |  | [0.006,0.126] | [0.060,0.368] | [0.063,0.174] | [-0.446,-0.104] |
| Y2012Q4 |  | 0.037 | 0.228* | 0.089*** | -0.120 |
|  |  | [-0.012,0.086] | [0.047,0.410] | [0.039,0.138] | [-0.270,0.031] |
| Y2013Q1 |  | 0.036 | 0.170* | 0.098* | -0.056 |
|  |  | [-0.017,0.089] | [0.023,0.317] | [0.016,0.180] | [-0.199,0.086] |
| Y2013Q2 |  | 0.049 | 0.140 | 0.094* | -0.113 |
|  |  | [-0.015,0.114] | [-0.017,0.296] | [0.023,0.166] | [-0.294,0.068] |
| Y2013Q3 |  | 0.110*** | 0.397*** | 0.141*** | 0.022 |
|  |  | [0.052,0.167] | [0.238,0.555] | [0.062,0.219] | [-0.132,0.176] |
| Y2013Q4 |  | 0.013 | 0.255** | 0.076 | 0.086 |
|  |  | [-0.053,0.078] | [0.097,0.414] | [-0.014,0.165] | [-0.074,0.245] |
| Illness Burden |  | 0.010*** | -0.003*** | 0.005*** | -0.003*** |
|  |  | [0.009,0.010] | [-0.004,-0.003] | [0.005,0.005] | [-0.004,-0.002] |
| Age 19-29 |  | -0.161** | -3.242*** | 0.856*** | -0.623*** |
|  |  | [-0.281,-0.040] | [-4.106,-2.378] | [0.793,0.920] | [-0.917,-0.329] |
| Age 30-39 |  | -0.197*** | -24.563*** | 0.510*** | -0.378** |
|  |  | [-0.279,-0.115] | [-25.374,-23.753] | [0.463,0.556] | [-0.605,-0.152] |
| Age 40-49 |  | -0.164*** | -0.705*** | 0.334*** | -0.074 |
|  |  | [-0.216,-0.113] | [-0.846,-0.564] | [0.312,0.357] | [-0.177,0.028] |
| Male/Other |  | -0.622*** | -1.331*** | -0.018* | -0.209*** |
|  |  | [-0.655,-0.589] | [-1.477,-1.186] | [-0.037,0.000] | [-0.296,-0.121] |
| Risk |  | 0.051* | -0.018 | -0.005 | -0.190** |
|  |  | [0.011,0.091] | [-0.082,0.047] | [-0.046,0.036] | [-0.324,-0.057] |
| 1 Condition |  | -0.175*** | -0.406*** | 0.054** | -0.037 |
|  |  | [-0.208,-0.142] | [-0.490,-0.321] | [0.016,0.092] | [-0.145,0.072] |
| 2+ Conditions |  | -0.162*** | -0.809*** | 0.193*** | -0.056 |
|  |  | [-0.214,-0.110] | [-0.913,-0.705] | [0.125,0.260] | [-0.214,0.102] |
| Self |  | -0.227*** | -0.307*** | -0.026 | -0.183* |
|  |  | [-0.261,-0.193] | [-0.407,-0.208] | [-0.060,0.008] | [-0.348,-0.019] |
| Small Employer50 |  | -0.022 | 0.004 | -0.022 | -0.094 |
|  |  | [-0.059,0.015] | [-0.092,0.100] | [-0.055,0.012] | [-0.250,0.062] |
| _cons |  | -2.713*** | 1.974*** | -2.782*** | -0.564 |
|  |  | [-2.861,-2.564] | [1.762,2.186] | [-2.992,-2.573] | [-1.318,0.190] |
|  |  | 21,008,072 | 21,008,072 | 21,008,072 | 21,008,072 |

Two-part models controlling for: *Quarters by year, Age, Gender, # of Chronic Conditions, Illness Burden, Fully Insured Group, Dependent Status, Employer Size, County*

Coefficients with 95% confidence intervals in brackets

^*^ *p* < 0.05, ^**^ *p* < 0.01, ^***^ *p* < 0.001
