# Supplementary material for: The CareFirst Patient-Centered Medical Home Program: Cost and Utilization Effects in Its First Three Years
Source: J Gen Intern Med. 2016 Jul 29;31(11):1382–8. doi: 10.1007/s11606-016-3814-z (PMC5071295; doi:10.1007/s11606-016-3814-z)
Supplement: Supplementary file 5 — (DOCX 38 kb) [file 11606_2016_3814_MOESM5_ESM.docx]

**Appendix 5: Robustness Check including intermittent enrollees as PCMH Treatment Group**

|  |  | **I. Total Allowed Amount** | | **II. Inpatient Allowed Amount** | | **III. Emergency Department Allowed Amount** | | **IV. Prescription Drug Allowed Amount** | |
| --- | --- | --- | --- | --- | --- | --- | --- | --- | --- |
|  |  | **Logit Regression**  **(Part One)** | **Generalized Linear Model**  **(Part Two)** | **Logit Regression**  **(Part One)** | **Generalized Linear Model**  **(Part Two)** | **Logit Regression**  **(Part One)** | **Generalized Linear Model**  **(Part Two)** | **Logit Regression**  **(Part One)** | **Generalized Linear Model**  **(Part Two)** |
| PCMH |  | -0.002 | -0.086*** | -0.030 | -0.042** | 0.044* | -0.019* | 0.032* | -0.074*** |
|  |  | [-0.022, 0.018] | [-0.103, -0.068] | [-0.070, -0.009] | [-0.071, -0.014] | [-0.004, 0.092] | [-0.039, -0.000] | [-0.003, 0.066] | [-0.107, -0.041] |
| Post_Y1 |  | 0.061*** | -0.026 | -0.021 | -0.066*** | -0.012 | -0.002 | 0.088*** | -0.046* |
|  |  | [0.027, 0.095] | [-0.081, 0.029] | [-0.067, 0.025] | [-0.101, -0.031] | [-0.054, 0.029] | [-0.022, 0.018] | [0.043, 0.132] | [-0.096, -0.004] |
| Post_Y2 |  | -0.052*** | -0.124*** | -0.113** | -0.093*** | -0.105*** | -0.027** | -0.001 | -0.088*** |
|  |  | [-0.083, -0.021] | [-0.144, -0.104] | [-0.163, -0.063] | [-0.129, -0.056] | [-0.160, -0.051] | [-0.046, -0.007] | [0.042, 0.040] | [-0.128, -0.048] |
| Post_Y3 |  | -0.060*** | -0.162** | -0.117** | -0.120** | -0.148*** | -0.050*** | -0.055** | 0.129*** |
|  |  | [-0.095, -0.024] | [-0.188, -0.136] | [-0.179, -0.055] | [-0.178, -0.062] | [-0.224, -0.073] | [-0.076, -0.024] | [-0.110, 0.000] | [-0.168, 0.091] |
| Y2010Q2 |  | -0.170*** | 0.041*** | -0.033 | 0.055 | 0.048** | -0.022* | 0.015** | 0.009 |
|  |  | [-0.219, -0.121] | [0.025, 0.058] | [-0.017, 0.082] | [-0.015, 0.126] | [0.015, 0.080] | [-0.047, -0.003] | [0.001, 0.028] | [-0.003, 0.022] |
| Y2010Q3 |  | -0.453*** | 0.054*** | -0.036 | 0.136*** | 0.026 | 0.005 | -0.072*** | 0.024* |
|  |  | [-0.498, -0.409] | [0. 032, 0. 077] | [-0.094, 0.022] | [0.062, 0.210] | [-0.006, 0.058] | [-0.031, 0.041] | [-0.094, -0.050] | [-0.004, 0.053] |
| Y2010Q4 |  | -0.540*** | 0.093*** | -0.023 | 0.149*** | -0.045** | 0.020 | -0.065** | 0.028** |
|  |  | [-0.422, -0.337] | [0.073, 0.113] | [-0.074, 0.028] | [0.080, 0.218] | [-0.084, -0.005] | [-0.009, 0.049] | [-0.108, -0.021] | [0.007, 0.049] |
| Y2011Q1 |  | -0.379*** | 0.314*** | 0.047 | 0.273*** | 0.078*** | 0.068*** | 0.018 | 0. 078** |
|  |  | [-0.422, -0.337] | [0.188, 0.441] | [-0.014, 0.107] | [0.155, 0.391] | [0.042, 0.114] | [0.042, 0.094] | [-0.004, 0.040] | [0.018, 0.137] |
| Y2011Q2 |  | -0.126*** | 0.291*** | 0.016** | 0.282*** | 0.099*** | 0.047** | -0.004 | 0.114*** |
|  |  | [-0.161,- 0.092] | [0.159, 0.423] | [-0.039, 0.072] | [0.174, 0.389] | [0.062, 0.136] | [0.017, 0.077] | [-0.023, 0.014] | [0.050, 0.179] |
| Y2011Q3 |  | 0.092** | 0.363*** | 0.024 | 0.318*** | 0.156** | 0.091*** | -0.055*** | 0.148** |
|  |  | [0. 024, 0.161] | [0.195, 0.530] | [-0.047, 0.095] | [0.204, 0.433] | [0.109, 0.202] | [0.060, 0.122] | [-0.075,-0.034] | [0.074, 0.223] |
| Y2011Q4 |  | 0.047** | 0.176*** | -0.068** | 0.282*** | 0.067** | 0.077*** | -0.090*** | 0.075*** |
|  |  | [0.011, 0.084] | [0.070, 0.282] | [-0.122, -0.014] | [0.211, 0.353] | [0.027, 0.107] | [0.049, 0.105] | [-0.126, -0.054] | [0.021, 0.128] |
| Y2012Q1 |  | 0.100*** | 0.179*** | 0.000 | 0.174*** | 0.138*** | 0.117*** | -0.155*** | 0.099*** |
|  |  | [0.083, 0.116] | [0.141, 0.216] | [-0. 066, 0.066] | [0.084, 0.263] | [0.093, 0.183] | [0.088, 0.147] | [-0.180, -0.131] | [0.062, 0.137] |
| Y2012Q2 |  | 0.033** | 0.175*** | -0.025 | 0.174*** | 0.141*** | 0.154*** | -0.263*** | 0.109*** |
|  |  | [0.011, 0.055] | [0.152, 0.198] | [-0.080, 0.030] | [0.084, 0.263] | [0. 101, 0.181] | [0.126, 0.181] | [-0.289, -0.237] | [0.071, 0.147] |
| Y2012Q3 |  | 0.021* | 0.175** | -0.032 | 0.220*** | 0. 149*** | 0.164*** | -0.396*** | 0.094*** |
|  |  | [-0.003, 0.046] | [0.146, 0.204] | [-0.093, 0.029] | [0.145, 0.294] | [0. 099, 0.200] | [0.136, 0.193] | [-0.430, -0.362] | [0.046, 0.143] |
| Y2012Q4 |  | 0.058*** | 0.212*** | -0.047 | 0.225*** | 0. 106*** | 0.169*** | -0.396*** | 0.116*** |
|  |  | [0.036, 0.080] | [0.196, 0.228] | [-0.112, 0.018] | [0.144, 0.305] | [0. 056, 0.156] | [0.142, 0.197] | [-0.431, -0.362] | [0.071, 0.162] |
| Y2013Q1 |  | 0.076*** | 0.260*** | -0.038 | 0.309*** | 0. 125*** | 0.196*** | -0.373*** | 0.158*** |
|  |  | [0.033, 0.120] | [0.243, 0.277] | [-0.096, 0.019] | [0.240, 0.379] | [0. 054, 0.197] | [0.161, 0.230] | [-0.400, -0.346] | [0.131, 0.185] |
| Y2013Q2 |  | 0.033 | 0.290*** | -0.044 | 0.346*** | 0. 122** | 0.202*** | -0.409*** | 0.208*** |
|  |  | [-0.011, 0.076] | [0.261, 0.319] | [-0.121, 0.032] | [0.261, 0.431] | [0. 052, 0.193] | [0.174, 0.231] | [-0.436, -0.382] | [0.175, 0.240] |
| Y2013Q3 |  | 0.157*** | 0.226*** | -0.018 | 0.303*** | 0. 135*** | 0.262*** | -0.390*** | 0.217*** |
|  |  | [0.132, 0.182] | [0.180, 0.271] | [-0.099, 0.062] | [0.232, 0.374] | [0. 067, 0.204] | [0.233, 0.292] | [-0.421, -0.360] | [0.187, 0.247] |
| Y2013Q4 |  | 0.225*** | 0.208*** | -0.098** | 0.279*** | 0. 048 | 0.278*** | -0.373*** | 0.217*** |
|  |  | [0.198, 0.252] | [0.140, 0.276] | [-0.169, -0.026] | [0.206, 0.353] | [-0.032, 0.128] | [0.240, 0.316] | [-0.403, -0.342] | [0.160, 0.273] |
| Illness Burden |  | 0.005*** | 0.004*** | 0.011*** | 0.002*** | 0.006*** | 0.001*** | 0.003*** | 0.003*** |
|  |  | [0.005, 0.006] | [0.004, 0.005] | [0.010, 0.011] | [0.001, 0.002] | [0.005, 0.006] | [0.001, 0.001] | [0.003, 0.003] | [0.003, 0.003] |
| Age 19-29 |  | 0.162*** | 0.090*** | 0.703** | 0.178*** | 0.944*** | 0.115*** | 0.220*** | -0.136*** |
|  |  | [0.150, 0.173] | [0.074, 0.106] | [0.669, 0.738] | [0.123, 0.233] | [0.915, 0.974] | [0.097, 0.134] | [0.195, 0.244] | [-0. 165, -0.106] |
| Age 30-39 |  | 0.139*** | 0.139*** | 0.818*** | 0.071** | 0.557*** | 0.085*** | 0.157*** | -0.146*** |
|  |  | [0.126, 0.151] | [0.124, 0.154] | [0.792, 0.844] | [0.021, 0.121] | [0.533, 0.580] | [0.070, 0.101] | [0.130, 0.184] | [-0.169, -0.123] |
| Age 40-49 |  | 0.042*** | -0.012 | 0.118*** | -0.097*** | 0.335*** | 0.074*** | 0.080*** | -0.056*** |
|  |  | [0.030, 0.053] | [-0.031, 0.008] | [0.095, 0.140] | [-0.147, -0.046] | [0.319, 0.350] | [0.062, 0.085] | [0.062, 0.098] | [-0.079,-0.034] |
| Male/Other |  | -0.558*** | -0.014** | -0.228*** | 0.162*** | 0.011** | -0.055*** | -0.377*** | 0.183*** |
|  |  | [-0.574, -0.542] | [-0.025,-0.004] | [-0.251, -0.204] | [0.131, 0.192] | [-0.007, 0.028] | [-0.066, -0.043] | [-0.410, -0.343] | [0.148, 0.218] |
| Risk |  | 0.589*** | 0.038*** | -0.076*** | -0.047*** | 0.026 | 0.087*** | 2.721*** | -0.351*** |
|  |  | [0.544, 0.633] | [0.017, 0.060] | [-0.108, -0.044] | [-0.092, -0.002] | [-0.001, 0.054] | [0.070, 0.105] | [2.467, 2.974] | [-0.428,-0.274] |
| 1 Condition |  | 0.484*** | 0.122*** | -0.145*** | 0.082*** | 0.071*** | 0.064*** | 0.546*** | 0.156*** |
|  |  | [0.441, 0.527] | [0.088, 0.155] | [-0.169, -0.121] | [0.042, 0.122] | [0.052, 0.091] | [0.049, 0.079] | [0.526, 0.566] | [0.104, 0.208] |
| 2+ Conditions |  | 0.961*** | 0.395*** | 0.037** | 0.225*** | 0.224*** | 0.101*** | 0.880*** | 0.397*** |
|  |  | [0.899, 1.024] | [0.334, 0.456] | [0.005, 0.070] | [0.174, 0.277] | [0.190, 0.259] | [0.086, 0.116] | [0.852, 0.907] | [0.352, 0.442] |
| Self |  | 0.076*** | -0.044*** | -0.119*** | -0.015 | 0.006 | -0.041*** | 0.088*** | -0.047** |
|  |  | [0.067, 0.084] | [-0.053, -0.035] | [-0.134, -0.103] | [-0.049, 0.018] | [-0.007, 0.020] | [-0.053, -0.030] | [0.075, 0.188] | [-0.093,-0.001] |
| Small Employer50 |  | 0.166*** | 0.029*** | -0.054** | -0.032 | -0.001 | 0.020* | 0.556*** | 0.084*** |
|  |  | [0.120, 0.212] | [0.018, 0.041] | [-0.082, -0.025] | [-0.071, 0.006] | [-0.022, 0.021] | [-0.003, 0.037] | [0.427, 0.684] | [0.053, 0.116] |
| _cons |  | 0.899*** | 7.092*** | -3.929** | 8.444*** | -3.282*** | 6.525*** | -2.765*** | 6.450*** |
|  |  | [0.859, 0.940] | [7.062, 7.123] | [-4.013, -3.846] | [8.342, 8.546] | [-3.327, -3.238] | [6.500, 6.551] | [-2.955, -2.576] | [6.369, 6.531] |
|  |  | 24,641,055 | 24,641,055 | 24,641,055 | 24,641,055 | 24,641,055 | 24,641,055 | 24,641,055 | 24,641,055 |

Two-part models controlling for: *Quarters by year, Age, Gender, # of Chronic Conditions, Illness Burden, Fully Insured Group, Dependent Status, Employer Size, County*

Coefficients with 95% confidence intervals in brackets

^*^ *p* < 0.05, ^**^ *p* < 0.01, ^***^ *p* < 0.001
